# Identification of key environmental factors and intelligent ecological zoning in the upper Yangtze basin

**DOI:** 10.1016/j.isci.2026.116001

**Published:** 2026-05-20

**Authors:** Wentong Wang, Peng Hu, Yunzhong Jiang, Baolong Zhao, Qin Yang, Qinghui Zeng, Huasen Lu

**Affiliations:** 1 State Key Laboratory of Water Cycle and Water Security in River Basin, China Institute of Water Resources and Hydropower Research, Beijing 100038, China

**Keywords:** marine organism, ecology, freshwater aquaculture

## Abstract

Ecological zoning is indispensable for delineating heterogeneous environmental regimes and informing targeted basin management. To elucidate the drivers of phytoplankton community structure, we analyzed water chemistry and phytoplankton data from 26 sites in the upper Yangtze River using random forest modeling. We identified total nitrogen, total phosphorus, water temperature, and pH as primary determinants, enabling the delineation of three distinct ecological zones. These zones exhibit significant geographical variations in physical profiles and species richness. Niche analysis revealed a positive correlation between species niche breadth and overlap. Importantly, the nitrogen-to-phosphorus ratio emerged as the critical regulator of spatial differentiation and niche characteristics. Regulating these nutrient ratios is vital for maintaining ecosystem stability. This predictive framework clarifies the mechanisms shaping phytoplankton distribution and provides a quantitative basis for region-specific conservation and nutrient-management strategies in large river systems.

## Highlights

• Random forest modeling identifies key drivers of phytoplankton distribution
• Unsupervised clustering divides the upper Yangtze River into three zones
• Phytoplankton niche breadth correlates positively with niche overlap
• N:P ratios regulate community composition and spatial distribution

## Graphical abstract

**Figure:**
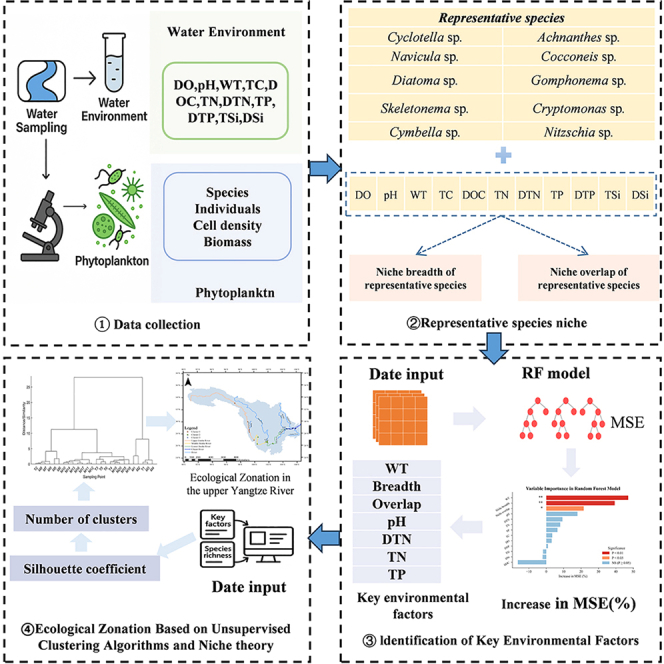

## Introduction

As important primary producers, phytoplankton are widely distributed in freshwater bodies such as lakes and rivers.^1^^,^^2^ Through photosynthesis, they utilize sunlight, carbon dioxide, and water to produce organic matter, providing food for higher organisms, such as fish, crustaceans, and mollusks. They form the foundation of the food chain and supply oxygen essential for the metabolism of most aquatic organisms.^3^^,^^4^^,^^5^^,^^6^ Additionally, owing to their small size, short lifespan, and rapid generational turnover, phytoplankton serve as important indicator species for monitoring the health of aquatic ecosystems.^7^^,^^8^^,^^9^ They participate in the material cycle, energy flow, and information transfer within aquatic ecosystems^10^ and exhibit high sensitivity to changes in water environmental factors, particularly in terms of species composition, community dynamics, and diversity variations.^11^ Changes in the diversity and community structure of phytoplankton are key factors for maintaining the stability of aquatic ecosystems.^12^^,^^13^^,^^14^ Moreover, diverse species compositions play an important role in withstanding environmental pressures such as shifts in temperature, nutrient levels, and pH.^6^

With the intensified impacts of global climate change and human activity in recent years, the accumulation of nutrient discharge loads has led to water eutrophication, resulting in a sharp increase in the frequency, scale, and duration of harmful algal blooms worldwide, particularly in lakes, reservoirs, and other stagnant water environments.^15^^,^^16^^,^^17^ Research on freshwater phytoplankton has primarily focused on eight phyla,^18^^,^^19^^,^^20^ namely *Bacillariophyta, Pyrrophyta, Chrysophyta, Cyanophyta, Euglenophyta, Chlorophyta, Cryptophyta, and Xanthophyta*. Research on phytoplankton niches has focused primarily on niche breadth and overlap. Species within a community evolve to utilize different portions of resource gradients (e.g., light intensity and nutrients) to reduce niche overlap and competition to reduce niche overlap and competition.^21^ The distribution of phytoplankton species and community structure characteristics results from the combined effects of multiple environmental factors such as physical parameters, nutrients, and metal ions.^22^^,^^23^^,^^24^^,^^25^ Nutrients are considered the primary drivers of phytoplankton community succession.^26^ However, the mechanisms underlying the interactions between phytoplankton community structure and environmental factors remain unclear. Most existing studies have overlooked the relationship between phytoplankton niches and water chemistry indicators. A deeper exploration of this relationship is crucial for understanding how phytoplankton populations respond to environmental changes and for providing theoretical support for ecosystem restoration.^27^^,^^28^

**Figure 1:**
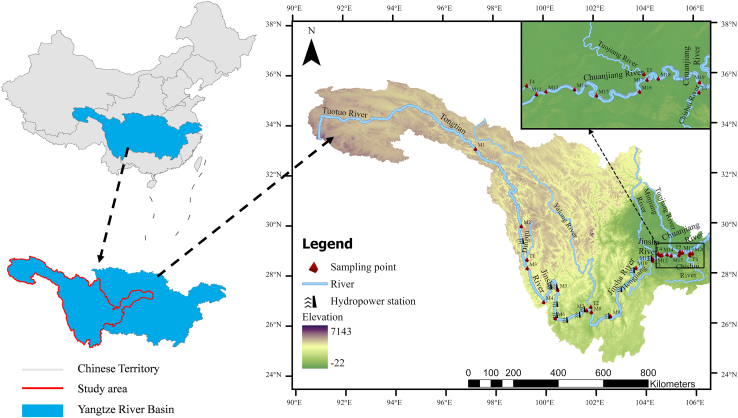
Distribution of the study area and sampling points in the upper Yangtze River Basin See also Table S1.

Since 1950, over 50,000 dams have been built across the Yangtze River Basin in China to control flooding, generate electricity, support irrigation, and store water.^29^ The construction of dams has provided significant socioeconomic benefits, such as flood control, irrigation, hydroelectric power generation, and improved navigation, providing crucial support for regional economic growth and energy supply. However, dam construction significantly affects the ecosystems, including alterations to phytoplankton communities.^30^ By modifying river hydrology, nutrient distribution, and sedimentation patterns, dams often cause significant changes in the structure and diversity of phytoplankton communities in water bodies.^30^^,^^31^ Additionally, dams alter the water temperature (WT), light conditions, and hydrodynamics, further influencing the species composition and community dynamics of phytoplankton.^32^ Therefore, phytoplankton have become a key biological indicator in ecological studies of the upper Yangtze River Basin. The upper Yangtze River Basin is located between 90°13′–111°30′E and 24°37′–35°54′N. It covers an area of 1.006 × 10^6^ km^2^, accounting for 55.9% of the total Yangtze River Basin area. Its total length is approximately 4,500 km, representing 71.4% of the river’s length.^33^ The total elevation drop exceeds 5,100 m, constituting 95% of the Yangtze River’s total drop. The annual average temperature in the upper Yangtze River region exhibits a pattern of higher temperatures in the east and south and lower temperatures in the west and north, with the river source area having the lowest temperature. Owing to topographical influences, several enclosed temperature centers have formed, including the Sichuan Basin, Yunnan-Guizhou Plateau, and Jinsha River Valley. The mainstream and tributaries of the upper Yangtze River typically exhibit mountainous river geomorphology characterized by narrow valleys with V-shaped or U-shaped cross-sections, steep longitudinal slopes, and fast-flowing water, often forming deep pools. The riverbanks feature multiple terraces, and the region experiences significant elevation differences, steep gradients, and high sinuosity coefficients. Mountains and hills account for approximately 98% of the area of the upper Yangtze River Basin. This unique topography results in diverse climates, including alpine plateau, subtropical monsoon, and humid subtropical monsoon climates, with low temperatures, floods, and droughts being the primary climatic hazards.^34^ The general distribution of hydropower resources in the Yangtze River Basin follows a pattern of abundance in the west and scarcity in the east, with the upper Yangtze region above Yichang containing approximately 80% of its hydropower potential. The upper reaches of the Yangtze River, from its source to Yichang, are sequentially called the Jinsha River and the Chuan River, with the confluence of the Min River serving as the boundary. The upstream section is referred to as the Jinsha River, while the downstream section is known as the Chuan River. The Jinsha River, located in the upper Yangtze River, is China’s largest hydropower base, with an estimated hydroelectric potential of approximately 112 million kW. At present, 12 hydropower stations have been constructed in the main stream of the upper Yangtze River, with 6 under construction and 8 planned. Field surveys were mainly conducted along the upper mainstream, with sampling sites (M1–M20) strategically placed downstream of the key hydropower dams. Because tributaries are important sources of nutrients, the study included major tributaries of the upper Yangtze River Basin, such as the Dingqu, Yalong, Heng, Min, and Chishui Rivers, with additional sampling sites (T1–T6) set up at their confluence with the mainstream (Figure 1).

Current zoning studies on the upper Yangtze River focus mostly on natural watershed boundaries, lacking ecologically based divisions and detailed analyses of ecological characteristics. This has hindered the development of targeted management and restoration strategies.^35^^,^^36^ To address this gap, our study had three objectives: (1) to examine the characteristics of phytoplankton communities in the upper Yangtze River, (2) to identify key environmental factors influencing these communities, and (3) to establish a scientific ecological zoning of the region to provide a theoretical basis for ecosystem protection and restoration. To achieve these objectives, this study employed the following analytical approaches: (1) screening for dominant phytoplankton species in key areas of the upper Yangtze River, and calculating niche characteristics and species richness of the communities; (2) using a random forest (RF) model to qualitatively assess and identify key environmental factors affecting phytoplankton communities; and (3) applying an unsupervised machine learning algorithm to cluster sites based specifically on the identified key environmental factors, thereby performing scientific ecological zoning that translates biological-environmental relationships into spatial management units, and analyzing the geographic distribution characteristics to support and guide watershed ecological restoration efforts. In summary, this work uncovers the main drivers and mechanisms behind phytoplankton community shifts, establishes a data-driven ecological zoning framework for the upper Yangtze River, and provides theoretical support for precise restoration strategies aimed at preserving aquatic biodiversity and ecosystem stability.

## Results

### Phytoplankton community structure and representative species selection

**Figure 2:**
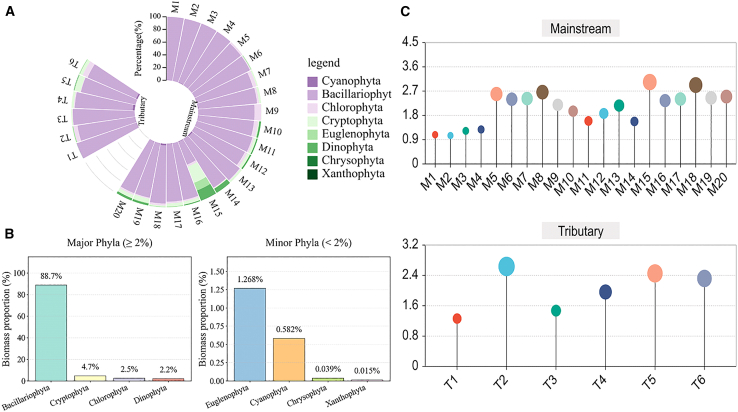
Phytoplankton community composition and the magnitude of species richness in the study area (A) Percentage composition of phytoplankton species at each sampling site. (B) Proportion of different phytoplankton species across all sampling sites in the study area. (C) Richness of species at each sampling point. See also Table S2.

The planktonic community composition of the study area is shown in Figure 2. A total of 89 phytoplankton species from 8 phyla were detected in the study area, namely 12 Cyanophyta, 31 Bacillariophyta, 31 Chlorophyta, 4 Cryptophyta, 3 Euglenophyta, 3 Dinophyta, 4 Chrysophyta, and 1 Xanthophyta. Bacillariophyta were the dominant group in the phytoplankton community, accounting for 88.74% of the total biomass. Cryptophyta accounted for 4.73% of the total abundance, followed by Chlorophyta (2.48%), Dinophyta (2.15%), Euglenophyta (1.27%), Cyanophyta (0.58%), Chrysophyta (0.039%), and Xanthophyta (0.015%). Both tributaries and main channels in the upstream regions had simpler species compositions than the downstream areas, with diatoms (*Bacillariophyta*) accounting for >90% of the community. In the middle and lower reaches, diatoms maintained absolute dominance; however, the overall composition of algae became more diverse, with noticeable increases in the proportions of *Chlorophyta*, *Dinophyta*, and *Cryptophyta*.

In addition, significant differences in phytoplankton species richness were observed among the upper, middle, and lower reaches. In the upstream section, species richness initially increased, reaching a relatively high value. In the middle reaches, species richness remained relatively stable. In the downstream region, species richness initially declined, followed by an increase.

**Figure 3:**
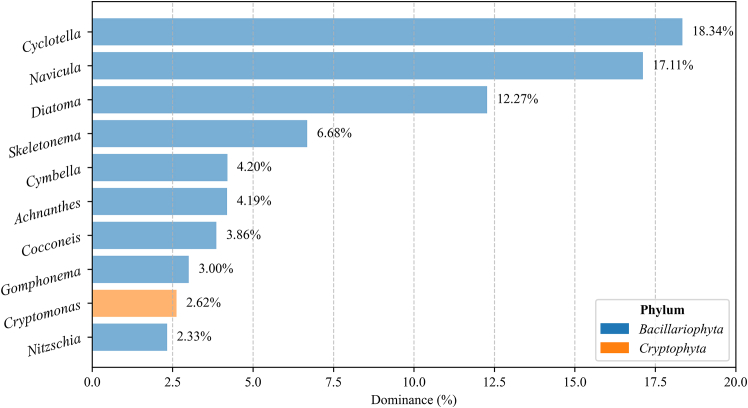
Dominance of representative species Data represent the McNaughton Dominance Index (Y) of the top ten dominant phytoplankton species.

The species dominance calculations are shown in Figure 3. Ten species with a dominance >2% were selected (Figure 3), including nine species from *Bacillariophyta* and one from *Cryptophyta*. Based on the dominance index, in descending order, these species were *Cyclotella, Navicula, Diatoma, Skeletonema, Cymbella, Achnanthes, Cocconeis, Gomphonema*, *Cryptomonas, and Nitzschia*. *Cyclotella* had the highest dominance at 18.34, followed by *Navicula* at 17.11 and *Nitzschia* at 2.33.

### Representative species niche

Niche breadth calculations for representative species under the 11 resource gradients are presented in Table 1. Mean-S refers to the average niche breadth of each species across all resource gradients, while Mean-R denotes the average niche breadth of all species under each specific gradient. *Gomphonema* exhibited the highest average niche breadth (4.35), whereas *Achnanthes* had the lowest, suggesting lower adaptability to environmental variation. Among the 10 representative species in the study area, the highest average niche breadth was observed along the TSi and DSi concentration gradients, whereas the lowest was found along the WT gradient. This suggests that fluctuations in TSi have limited effects on community stability, while changes in WT strongly affect it. Most species exhibited optimal performance within the 21°C–21.6 °C range, indicating that temperature fluctuations are a major driver of community instability. Additionally, the niche breadths of TC, TP, and DTP were relatively low, indicating that variations in these nutrient concentrations could lead to dramatic changes in the phytoplankton community.^37^

**Table 1:** Niche breadth of representative species along 11 physicochemical parameter gradients

Species	DO	pH	WT	TC	DOC	TN	DTN	TP	DTP	TSi	DSi	Mean-S	Rank
*Gomphonema*	4.19	4.29	4.45	4.24	4.47	4.41	4.39	4.58	4.18	4.25	4.38	4.35	1
*Navicula*	3.84	4.53	4.12	3.59	4.72	4.13	4.11	3.96	3.87	4.50	3.89	4.11	2
*Cyclotella*	3.68	3.11	3.54	4.24	4.04	4.28	4.43	3.64	4.49	3.67	3.89	3.91	3
*Skeletonema*	3.43	2.74	2.89	3.67	3.29	3.90	3.88	3.77	4.30	4.94	4.89	3.79	4
*Nitzschia*	3.75	4.19	3.56	3.76	4.15	3.33	3.23	3.86	4.11	3.78	3.49	3.75	5
*Cymbella*	3.24	3.33	2.11	2.82	3.01	3.90	3.90	3.18	3.13	4.00	4.08	3.34	6
*Cryptomonas*	2.81	2.69	2.15	2.53	2.89	2.72	2.73	2.58	3.06	3.23	2.82	2.75	7
*Diatoma*	2.60	2.18	1.22	1.85	2.69	3.22	3.32	2.06	2.08	2.94	3.17	2.49	8
*Cocconeis*	3.15	3.13	2.43	1.91	2.75	2.05	2.05	2.85	2.58	2.36	1.97	2.47	9
*Achnanthes*	1.84	1.82	2.58	2.09	2.46	2.41	2.40	2.53	2.49	2.48	2.11	2.29	10
Mean-R	3.25	3.20	2.91	3.07	3.45	3.43	3.45	3.30	3.43	3.62	3.47	–	–
RANK	8	9	11	10	3	5	4	7	6	1	2	–	–

Mean-S refers to the average niche breadth of each species across all resource gradients. Mean-R denotes the average niche breadth of all species under each specific gradient.

**Figure 4:**
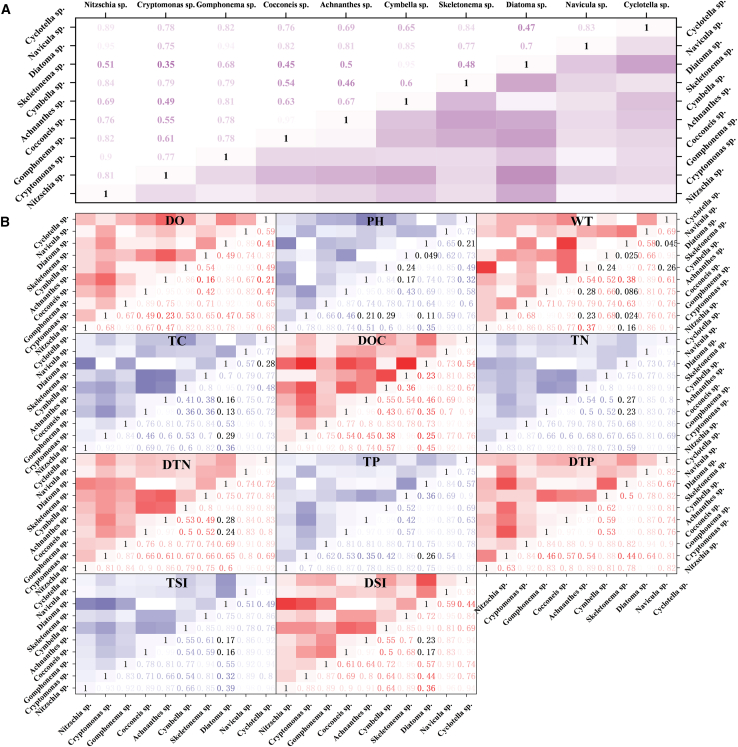
Mean niche overlaps between the representative species (A) Average ecological niche overlap matrix of different phytoplankton species. (B) Ecological niche overlap of different phytoplankton species under different environmental factors.

Niche overlap calculations for representative species under the 11 resource gradients are shown in Table 2. *Navicula* exhibited the highest niche overlap, followed by *Gomphonema*, whereas *Diatoma* had the lowest niche overlap. This indicates that *Navicula* has the greatest similarity in resource utilization with other species, whereas *Diatoma* has the least similarity.^38^ The niche overlap values along the DTP, TP, TN, and DTN gradients were the highest, indicating that the phytoplankton in the study area exhibited similar nitrogen and phosphorus utilization behaviors. Species preferences for nitrogen and phosphorus concentrations are concentrated within the same range, which may lead to intensified competition among species under resource-limited conditions. In contrast, the pH and WT gradients had the lowest niche overlap value (5.72), suggesting a more distinct differentiation in the species concentration requirements for these factors, resulting in lower direct competition among species. At the individual species level, *Navicula* exhibited the greatest overlap with *Nitzschia*, *Nitzschia* had the greatest overlap with *Navicula*, and *Nitzschia* had the greatest overlap with *Cyclotella* (Figure 4A). Niche overlap varied across environmental gradients, reflecting differential competition for shared ecological resources.

**Table 2:** Niche overlap of representative species along 11 physicochemical parameter gradients

Species	DO	pH	WT	TC	DOC	TN	DTN	TP	DTP	TSi	DSi	Mean-S	Rank
*Navicula*	7.18	6.98	6.96	7.13	7.33	7.73	7.72	7.39	7.70	7.77	7.68	7.41	1
*Gomphonema*	7.46	6.81	6.93	7.35	7.52	7.28	7.27	7.15	7.92	6.90	7.45	7.28	2
*Nitzschia*	6.84	6.51	6.54	6.95	7.23	7.50	7.44	7.46	7.49	7.37	7.45	7.16	3
*Cyclotella*	5.24	5.36	5.72	6.29	7.42	7.52	7.54	6.89	7.15	7.24	7.61	6.73	4
*Cocconeis*	6.60	6.49	6.08	5.10	6.12	6.17	6.17	7.33	7.25	6.30	6.46	6.37	5
*Cymbella*	7.09	5.90	4.42	5.67	5.58	6.81	6.80	7.23	7.62	6.31	6.34	6.34	6
*Achnanthes*	5.16	5.22	6.37	5.57	6.08	6.22	6.21	6.82	7.38	6.61	6.45	6.19	7
*Skeletonema*	5.41	4.76	5.40	6.15	6.14	6.34	6.31	5.84	6.42	7.43	7.13	6.12	8
*Cryptomonas*	5.15	4.83	5.88	5.98	5.78	6.45	6.43	5.22	5.82	6.84	6.49	5.90	9
*Diatoma*	6.73	4.38	2.91	3.97	4.70	5.58	5.60	6.44	6.99	4.45	4.24	5.09	10
MEAN-R	6.29	5.723	5.720	6.02	6.39	6.76	6.75	6.78	7.17	6.72	6.73	–	–
RANK	8	10	11	9	7	3	4	2	1	6	5	–	–

Values represent Pianka’s niche overlap index between representative species across different resource gradients.

In the dissolved oxygen (DO) gradient, the niche overlap index between *Cymbella* and *Diatoma* was 0.98, indicating that their DO resource-use demands are highly similar. This substantial overlap suggests that *Cymbella* and *Diatoma* may compete intensely for oxygen resources in similar ecological niches. In the pH gradient, the niche overlap index between *Cryptomonas* and *Skeletonema* was 0.96, showing that their pH resource-use demands are comparable. Such similarity implies that *Cryptomonas* and *Skeletonema* likely occupy overlapping habitat conditions with respect to pH, resulting in significant interspecific competition. In the total phosphorus (TP) gradient, the niche overlap index between *Achnanthes* and *Diatoma* was 0.99, indicating nearly identical TP resource-use demands. This exceptionally high overlap suggests that *Achnanthes* and *Diatoma* not only share similar nutrient preferences but may also experience strong competitive exclusion or coexistence pressure under phosphorus-limited conditions. In the total nitrogen (TN) gradient, the niche overlap index between *Cocconeis* and *Achnanthes* was 0.98, reflecting similar TN resource-use demands. Given this high degree of overlap, *Cocconeis* and *Achnanthes* are likely to engage in evident and potentially sustained competition when nitrogen availability becomes a limiting factor in their shared environment (Figure 4B).

Niche characteristics of the phytoplankton community in the study area revealed the varying effects of different environmental factors on species niches. TP and DTP exhibited the highest niche overlap and a relatively small niche breadth, indicating that phytoplankton have strict concentration requirements for these two factors with a highly concentrated demand, which may lead to intense competition among species. In contrast, TSi and DSi showed a lower niche overlap and larger niche breadth, suggesting that phytoplankton have a broader range of silicon requirements and experience less competition. TC displayed low niche breadth and overlap. Although these species had a narrow preference range for TC, their distribution was relatively uniform, leading to minimal competition. Overall, the high niche overlap and small niche breadth for nitrogen and phosphorus elements suggest that phytoplankton have concentrated demands for these nutrients, and fluctuations in their concentrations could cause significant community shifts. In contrast, silicon and carbon exhibited lower niche overlap and greater niche breadth, indicating that variations in these factors had a relatively smaller impact on the community. Additionally, DO, pH, and WT had smaller niche breadths and overlaps than nutrient elements, implying that phytoplankton have stricter requirements for these environmental factors. However, owing to the greater variation in species-specific preferences for these indicators, direct competition among species remains low.

**Figure 5:**
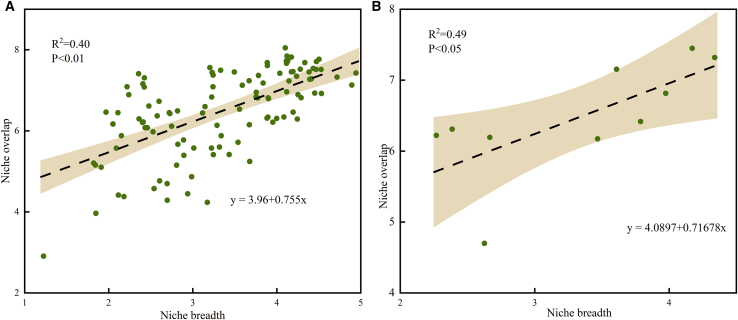
Linear regression analysis of niche breadth and niche overlap (A) Correlation between niche breadth and overlap for individual phytoplankton species based on 11 physicochemical indicators. (B) Correlation between average niche breadth and average overlap for dominant phytoplankton species. Statistical significance is evaluated using linear fitting (*p* values).

A significant positive correlation was observed between the average niche breadth of dominant species and their niche overlap (R^2^ = 0.40, *p* < 0.01; Figure 5). Specifically, *Navicula* and *Gomphonema* exhibited the highest niche overlaps (7.41 and 7.28, respectively), corresponding with the largest niche breadths (4.11 and 4.35, respectively). In contrast, *Diatoma* showed the lowest niche overlap among the dominant taxa, with its niche breadth ranking eighth. Furthermore, the two most abundant taxa in the study area, *Cyclotella* and *Navicula* (dominance indices of 18.34 and 17.11), ranked among the top three in niche breadth and the top four in niche overlap. These results indicate that dominant taxa with broader niche breadths in the Upper Yangtze Basin tend to exhibit higher overlap with co-occurring species.

### Identification of key environmental factors

**Figure 6:**
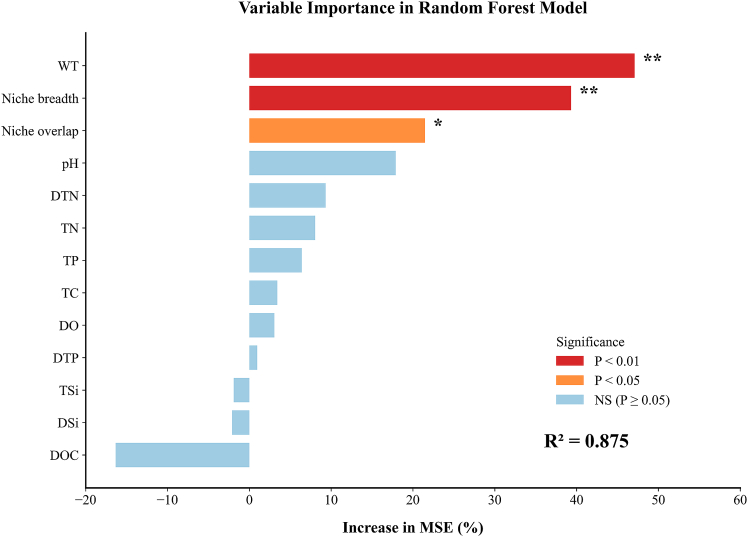
Variable importance ranking in the Random Forest model Importance ranking of environmental factors explaining species richness, measured by the increase in mean squared error (%IncMSE). The coefficient of determination (R^2^ = 0.875) indicates the model’s predictive performance.

To investigate the relative importance of environmental factors driving phytoplankton species richness, we constructed a RF model with species richness as the target variable. Input variables included WT, DO, nine nutrients, as well as niche breadth and niche overlap. Crucially, niche breadth and overlap serve as comprehensive indicators reflecting the intensity of interspecific competition and the adaptive capacity of organisms. By capturing signals of biological interactions that cannot be characterized by abiotic factors alone, these metrics significantly enhance the explanatory power of the model. Figure 6 shows the importance rankings of the environmental factors calculated using the RF model (R^2^ = 0.875). The key factors, in descending order of importance, were WT (47.09), Niche breadth (39.33), Niche overlap (21.47), pH (17.90), DTN (9.33), TN (8.05), TP (6.41), TC (3.42), DO (3.07), and DTP (0.97). WT, Niche breadth, and Niche overlap were significant (*p* < 0.05), with WT and Niche breadth exhibiting high significance (*p* < 0.01). Additionally, TSi (−1.90), DSi (−2.10), and DOC (−16.(34) showed negative values, indicating that when using %IncMSE to calculate variable importance, the permutation of these variables reduced the model’s MSE. This suggests that these variables contribute minimally to the model performance and may even introduce noise or redundant information, reducing the model’s predictive capability.^39^^,^^40^^,^^41^

To further explore the key factors that significantly influenced the target variable (species richness), we selected the seven indicators with the highest explanatory power for species richness based on the results of the RF regression model. These seven indicators, ranked by %IncMSE from highest to lowest, were WT (47.09), Niche breadth (39.33), Niche overlap (21.47), pH (17.90), DTN (9.33), TN (8.05), and TP (6.41). These indicators were highly important in the model, indicating that they are key factors affecting species richness variation, with WT and Niche breadth showing highly significant effects (*p* < 0.01) and Niche overlap reaching a significant level (*p* < 0.05). Other indicators, such as pH and DTN, which did not reach highly significant levels, still demonstrated explanatory power for species richness in the model. Additionally, by removing features that contributed less or even negatively to the target variable (e.g., TSi, DSi, and DOC), we retained features with greater contributions to the target variable while reducing noise and redundant features.^42^^,^^43^

### Ecological zoning and differences in environmental factors

**Figure 7:**
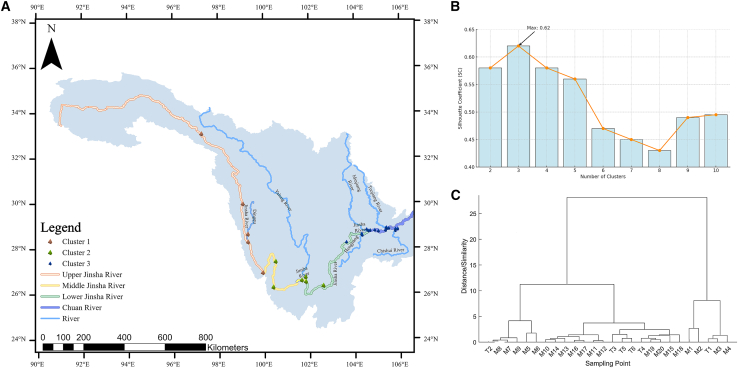
Clustering for unsupervised machine learning (A) Spatial distribution of different classifications across the study area. (B) Silhouette coefficient analysis for varying cluster numbers. (C) Hierarchical clustering dendrogram of sampling points.

The seven key environmental factors identified by the RF model, in conjunction with phytoplankton species richness, were utilized as the multivariate input matrix for hierarchical clustering to delineate functional ecological zones. The results of the unsupervised clustering indicate that when the number of clusters was set to three, the silhouette coefficient reached its maximum value (s Figure 7). The 26 sampling sites were divided into three clusters of 5, 6, and 15 sites. The Kruskal-Wallis test result was *p* = 0.02 < 0.05, indicating significant differences between the clusters and demonstrating good clustering performance.^44^ Furthermore, by restoring the geographical distributions of these three clusters, we found that their spatial distributions exhibited significant differences (Figure 7). Based on previous studies,^45^ it was observed that the three clusters identified through key environmental factors combined with unsupervised learning methods correspond geographically to the upper Jinsha River, the middle and lower reaches of the Jinsha River, and other main tributary regions in the upper Yangtze River connected to the lower Jinsha River. Specifically, the sites of Cluster 1 were mainly located in the main stream and major tributaries of the upper reaches of the Jinsha River, Cluster 2 sites were mainly located in the main stream and major tributaries of the middle and lower reaches of the Jinsha River, and Cluster 3 sites were mainly located in the main stream and major tributaries of the Chuanjiang River.

**Figure 8:**
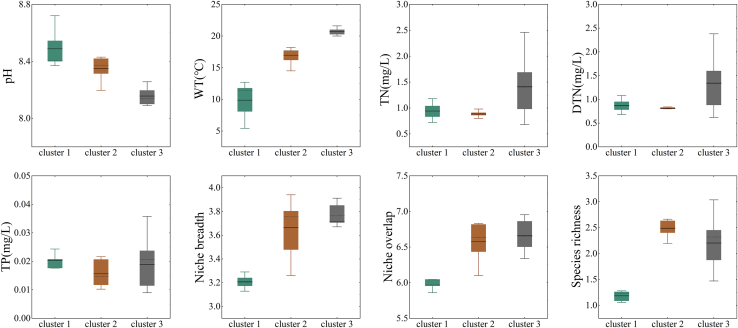
Size distribution of key environmental factors in different clusters Data are represented as boxplots show the median, Average, interquartile range, and whiskers (min/max) for environmental and ecological metrics across the three clusters.

**Figure 9:**
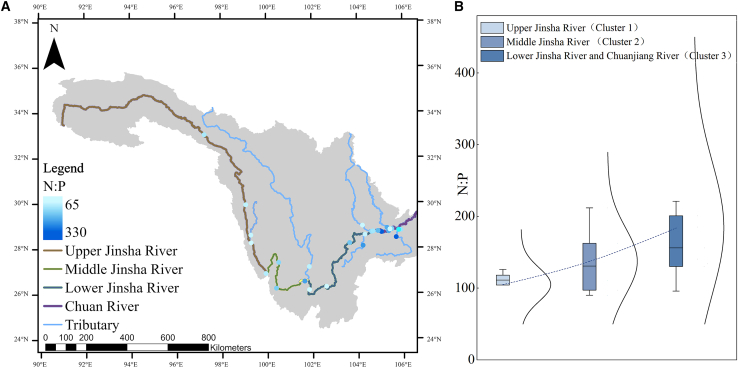
Spatial distribution of nitrogen-to-phosphorus (N:P) ratios in the study area (A) Distribution map of N:P ratios in the upper reaches of the Yangtze River. (B) Comparison of N:P ratios across the three ecological zones and between mainstream and tributary sites. Data are represented as boxplots show the mean, median, interquartile range, and whiskers.

Based on the results of the cluster analysis, key environmental factors (WT, Niche breadth, Niche overlap, pH, DTN, TN, and TP), and species richness, we analyzed the environmental characteristics of Cluster 1, Cluster 2, and Cluster 3 regions in the study area (Figure 8). The distribution of WT in the study area is influenced by multiple factors, including changes in elevation, topography, and climate. The upper Jinsha River section (cluster 1), which has a high elevation and cold climate, has relatively low WT. As the elevation decreases in the middle and lower reaches (Cluster 2), the region enters a dry-hot river valley climate and the WT significantly increases. The inflow of tributaries such as the Yalong River alters the thermal balance and runoff composition of the main stream, further increasing its WT. Various factors collaborate to cause the observed phenomenon of progressively increasing WT from the Cluster 1 to Cluster 3 region^46^. The upper Jinsha River is rich in carbonate rocks (such as calcite and dolomite) and feldspar minerals (such as plagioclase and potassium feldspar). These minerals release alkaline substances (such as carbonate ions) through weathering and dissolution, playing an important buffering role in the water and significantly increasing the pH. Although the middle and lower reaches of the Jinsha River and the Chuanjiang River section of the upper Yangtze River (Cluster 2, Cluster 3)are affected by human activities, such as agricultural and industrial emissions, the pH value slightly decreases because of the river’s self-purification capacity and dilution effect; however, it remains weakly alkaline.^47^ TN, DTN, and TP first decreased and then increased from the Cluster 1 to Cluster 3 region. Owing to the retention and interception effects of the cascade reservoirs, the TN, DTN, and TP gradually decreased from the upper Jinsha River to the middle and lower reaches. However, the concentrations gradually increased again owing to the inflow of the Min, Chishui, Tuotu, and Hengjiang River tributaries, as well as the increase in human activities.^45^^,^^48^ Furthermore, the spatial patterns of N:P stoichiometry across the basin provide additional corroboration for the ecological zoning. As shown in Figure 9, the N:P ratio consistently exceeded 50 throughout the study area, exhibiting a clear increasing trend from the upper Jinsha River to the Chuanjiang River (Figure 9). This longitudinal gradient is highly consistent with the spatial distribution of the identified ecological zones, indicating that the zoning framework effectively captures the fundamental shifts in nutrient structure and potential phosphorus limitation patterns from upstream to downstream. In terms of niche, the niche breadth and overlap in the study area showed a gradual increasing trend from the Cluster 1 to Cluster 3 region, being significantly lower in the upper Jinsha River region than in the middle and lower reaches of the middle and lower reaches region of the Jinsha River and the Chuanjiang section region (clusters 2 and 3). This indicates that the phytoplankton community in the upper Jinsha River region shows a lower degree of preference for overall resource status and experiences less competition. In the Cluster 2 and Cluster 3 regions, environmental factors were more favorable for the survival of the phytoplankton community, resulting in a higher degree of overall resource preference and competition, leading to a higher niche breadth and overlap compared to the Jinsha River. Phytoplankton species richness in the Cluster 1 region was lower than that in the Cluster 2 and Cluster 3 regions, which may be attributed to the high flow velocity, short hydraulic retention time, low WT, lack of appropriate nutrient concentrations, and the absence of suitable still-water zones for phytoplankton growth in the upper Jinsha River.^45^ Phytoplankton species richness in the water bodies from the Cluster 2 to the Cluster 3 region of the study area showed a decreasing trend. The main reason for this is the significant increase in human activities in this region compared to the middle and lower reaches of the Jinsha River, including agricultural emissions, industrial pollution, and accelerated urbanization processes, which have led to water quality deterioration, affecting river ecosystems and phytoplankton communities.

## Discussion

### Significance of nitrogen and phosphorus regulation in phytoplankton communities

Nitrogen and phosphorus are two key nutrients used for the biochemical functions of phytoplankton and are the primary limiting nutrients for phytoplankton growth in aquatic ecosystems.^49^^,^^50^^,^^51^ Their relative demand patterns are important for the management of phytoplankton communities and eutrophication.^52^ Balancing nitrogen and phosphorus inputs is particularly important across water bodies with varying nutrient statuses—both for controlling harmful algal blooms in eutrophic systems and enhancing primary productivity in oligotrophic waters.^53^^,^^54^

Building on this understanding, numerous studies have shown that nitrogen and phosphorus concentrations are key determinants of algal growth limitations under both natural and anthropogenic conditions.^52^ Regulating the TP and TN concentrations in water is an effective water quality management measure.^49^^,^^55^ Evidence from regions such as the United States, Australia, and China highlights the significant influence of nitrogen and phosphorus on phytoplankton community structure and overall ecosystem health.^56^^,^^57^^,^^57^^,^^58^^,^^59^^,^^60^^,^^61^

Maintaining a balanced nitrogen-to-phosphorus (N:P) ratio is essential for supporting phytoplankton diversity and stability.^62^^,^^63^ Excessively high or low N:P ratios can disrupt community composition: low N:P ratios (nitrogen excess) may promote cyanobacterial dominance, while high N:P ratios (phosphorus limitation) often favor Bacillariophyta and other non-cyanobacterial taxa.^64^ Such shifts influence ecological niches and interspecific competition, ultimately affecting community structure and ecosystem functioning.^65^

As shown in Figure 9, the N:P ratio in the study area consistently exceeded 50 and exhibited a clear increasing trend from the upper Jinsha River to the Chuanjiang River (Figure 9B). This sustained nutrient imbalance led to phytoplankton communities dominated by Bacillariophyta (diatom phylum) and characterized by low species diversity. The niche overlap indices under TP, DTP, TN, and DTN gradients were 6.78, 7.17, 6.76, and 6.75, ranking highest among the 11 biotic elements, while the corresponding niche breadth values (3.30, 3.43, 3.43, and 3.(45) were relatively low. Additionally, among the nutrients, DTN, TN, and TP were the top three contributors to phytoplankton species richness. These results indicate that nitrogen and phosphorus significantly influence niche characteristics and species richness, highlighting the practical importance of managing their inputs to improve community structure and support ecosystem restoration.

### An intelligent ecological zoning framework integrating biotic interactions and environmental gradients

Conventional ecological zoning methods primarily depended on large-scale remote sensing and geospatial datasets, such as vegetation indices, land use classifications, topographic features, and climatic gradients.^66^^,^^67^^,^^68^^,^^69^ These approaches typically involved substantial data acquisition, preprocessing, and spatial interpolation, with a predominant focus on abiotic heterogeneity,^70^^,^^71^ while overlooking critical biological processes and interspecific dynamics—particularly community-level competition and mechanisms of species coexistence. Critically, ecological interactions among species—such as competition, coexistence, and niche differentiation—along with niche-based functional traits, have rarely been incorporated into the zoning logic, resulting in spatial divisions that may lack ecological realism and biological interpretability.

To address these issues, we integrated niche theory with machine learning methods to quantify species-environment relationships and to derive ecological zones. Specifically, we applied a RF algorithm to identify the key environmental drivers of phytoplankton richness and community structure, using mean squared error (MSE)-based feature importance. Key drivers identified included TN, TP, dissolved nitrogen (DTN), WT, pH, and niche breadth and overlap. Compared to traditional methods such as principal component analysis (PCA) and canonical correspondence analysis (CCA), RF does not rely on linear assumptions and is better suited to capture complex, nonlinear interactions between species and environmental variables. Additionally, RF provides a more robust and interpretable ranking of variable importance, enhancing the precision of key factor identification in heterogeneous ecological systems.

We integrated niche theory with machine learning methods to quantify species-environment relationships and to derive functional ecological partitions. Specifically, we applied a RF algorithm to identify the key environmental drivers of phytoplankton richness and community structure, using MSE-based feature importance. Key drivers identified included TN, TP, DTN, WT, pH, and niche breadth and overlap.

**Figure 10:**
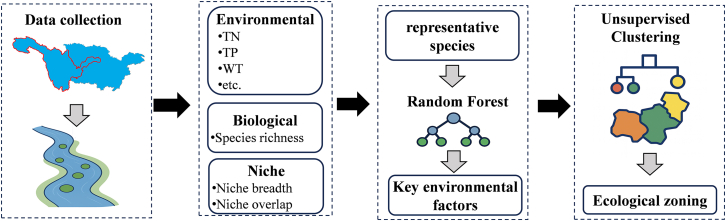
An intelligent ecological zoning framework integrates biotic interactions and environmental gradients

To translate these findings into spatially meaningful units, we applied an unsupervised hierarchical clustering algorithm to the key environmental variables identified by the RF model, along with species richness, niche breadth, and niche overlap (Figure 10). Using the study area as an example, this approach classified the 26 sampling sites into three ecologically coherent zones, each characterized by consistent patterns of species coexistence, community composition, and trophic structure. Notably, this high-resolution ecological zoning was achieved solely through targeted *in situ* sampling data, without reliance on traditional remote sensing inputs, and with explicit consideration of interspecific ecological interactions, thereby providing a robust foundation for localized and adaptive ecosystem management.

### Limitations of the study

The RF model and clustering algorithms successfully identified critical physicochemical drivers, such as WT, TN, TP, and pH, alongside niche interactions. However, the explicit quantitative incorporation of complex hydrodynamic metrics altered by cascade dams—such as precise hydraulic retention time, flow velocity profiles, and light attenuation—was limited.

The current ecological zoning framework is fundamentally anchored in phytoplankton communities as primary biological indicators. Future zoning efforts could benefit from integrating multi-trophic data, including zooplankton grazing pressure and benthic macroinvertebrates, to construct a more holistic representation of aquatic ecosystem health and food-web dynamics.

## Resource availability

### Lead contact

Further information and requests for resources should be directed to and will be fulfilled by the lead contact, Qinghui Zeng (qhzeng1990@126.com).

### Materials availability

This study did not generate new unique reagents or materials.

### Data and code availability

**Data:** The datasets supporting this study, including sampling locations and phytoplankton species lists, are provided in the supplemental information. Additional raw data are available from the lead contact upon reasonable request.

**Code:** This paper does not report original code. Analyses were performed using standard packages in R (v.4.4.1).

**Other items:** Any additional information required to reanalyze the data reported in this paper is available from the lead contact upon request.

## Acknowledgments

This work was supported by the National Key Research Program of China (grant no. 2024YFC3210901), the 10.13039/501100001809National Nature Science Foundation of China (grant no. 52394233, U2240202), the Independent Research Project of the State Key Laboratory of Water Cycle and Water Security (grant no. WR110146B0022024, SKL2025TDGG06, SKL2025RCPY09), and the Basic Scientific Research Expense Project of 10.13039/501100004614IWHR (grant no. WR110145B0102025).

## Author contributions

W.W.: investigation, methodology, writing – original draft, and data curation; P.H.: conceptualization, supervision, and writing – review and editing; Y.J.: supervision and writing – review and editing; B.Z., Q.Y., and H.L.: investigation and writing – review and editing; Q.Z.: conceptualization, supervision, validation, and writing – review and editing. All authors have read and agreed to the published version of the manuscript.

## Declaration of interests

The authors declare no competing interests.

## STAR★Methods

### Key resources table

**Table:** 

REAGENT or RESOURCE	SOURCE	IDENTIFIER
**Deposited data**		

Sampling locations and Phytoplankton species list	This paper	Tables S1 and S2

**Software and algorithms**		

R (v.4.4.1)	R Core Team	https://www.r-project.org/
Origin 2024	OriginLab	https://www.originlab.com/
randomForest R package	CRAN	https://cran.r-project.org/web/packages/randomForest/

**Other**		

Portable multiparameter water quality analyzer (YSI PRODSS)	YSI	https://www.ysi.com/prodss

### Method details

#### Data collection

To investigate the impact of water environmental factors on phytoplankton communities and to identify the key environmental factors affecting the ecological environment, from October to November 2023, we conducted measurements at 26 sampling sites in the upper Yangtze River (Figure 1). The measured parameters included dissolved oxygen (DO), pH, water temperature (WT), total carbon (TC), dissolved organic carbon (DOC), total nitrogen (TN), dissolved total nitrogen (DTN), total silicon (TSi), dissolved silicon (DSi), total phosphorus (TP) and dissolved TP (DTP). Additionally, we assessed the phytoplankton number of species, number of individuals, cell density, and biomass. Alkaline persulfate oxidation and phosphomolybdenum blue methods were used to measure TN, DTN, TP, and DTP concentrations.^72^ The TC and DOC were measured using a high-temperature combustion–nondispersive infrared detection method. TSi and DSi were determined using hydrofluoric acid conversion spectrophotometry and inductively coupled plasma emission spectroscopy. In addition, environmental and chemical parameters such as pH, DO, and WT were measured onsite using a portable multiparameter water quality analyzer(YSI PRODSS, OH, USA).

For phytoplankton samples, a 2 L water sample was collected and treated with 15 mL of Lugol’s solution. The sample was stirred to ensure that the phytoplankton was completely fixed. Finally, the treated water samples were sent to the laboratory for phytoplankton identification and analysis. Species identification was primarily based on “*Chinese Freshwater Algae – Systematics, Taxonomy, and Ecology*” and “*Common Freshwater Algae*”.^73^^,^^74^

#### Calculation methods

**Dominance assessment:**Dominance indicates the importance of a species, which can be used to assess community structure and aid management of aquatic ecosystems. In this study, representative species were determined using the McNaughton Dominance Index (Y),^75^ follows

$$Y=\frac{n_{i}}{N}\;f_{i}$$

where $n_{i}$ represents the number of individuals of the i-th phytoplankton species, N is the total number of all phytoplankton individuals, and $f_{i}$ denotes the occurrence frequency of the species across sampling sites. Species with Y ≥ 0.02 were selected as representative species.^76^

**Species richness:** Proposed by Spanish ecologist Margalef, Margalef's Richness Index is an ecological index used to measure the species richness within a community and is suitable for studying changes in species diversity within communities. This index has been widely applied in ecology, biodiversity research, and other related fields (Aslam et al., 2025; Kusuma et al., 2024; Ranglong et al., 2025). The calculation formula is^77^:

$$D=\frac{S-1}{lnN}$$

where S represents the number of species in the sample, and N is the total number of individuals in the sample.

**Niche characteristics:** Niche models use known species distribution data and relevant environmental variables to construct models that determine the ecological requirements of a species. To a certain extent, the niche reflects the spatial and temporal distribution, occupation, and utilization of the habitat by species and is influenced by biotic and environmental factors such as light, temperature, and nutrients.^78^ In recent years, niche models have been explored and applied in various fields of biodiversity conservation, and have been widely used to support conservation planning and ecosystem management.^79^^,^^80^ Niche breadth represents the total utilization of various resources by a species in its habitat, and is commonly used to reflect species distribution and resource occupation. A larger niche breadth indicates stronger competitiveness, whereas a smaller niche breadth suggests weaker competitiveness. Niche overlap reflects the competition and resource-sharing relationships between species. A high degree of niche overlap indicates similar resource demands among species.^81^^,^^82^ Generally, if two species exhibit a significant niche overlap, they have similar resource utilization behaviors, which may lead to intense competition under certain conditions. Species with broader niche breadths typically have stronger environmental adaptability, whereas those with narrower niche breadths are more sensitive to environmental changes. In resource-scarce situations, the former often have a greater survival advantage than the latter. However, when resources are relatively abundant, species with a narrower niche breadth, owing to their higher resource utilization efficiency, often demonstrate stronger competitiveness in localized habitats.^83^

There are various models for calculating niche breadth and overlap.^84^^,^^85^^,^^86^^,^^87^ This study adopts the widely used Levins' breadth and Pianka's overlap models to calculate niche breadth and overlap, respectively.

Levins' formula for calculating niche breadth (Bi)^85^ is as follows:

$$B_{i}=1/\sum_{j=1}^{R}{\left(P_{ij}\right)}^{2}$$

where, $P_{ij}$ is the ratio of the number of individuals of species i in resource state j to the total number of individuals of species i, and R is the total number of resource states. Resource states were divided into five intervals based on percentiles representing a gradient along a particular available resource. Available resources included TN, TP, DO, TSi, TC, etc.

Pianka’s formula for calculating niche overlap^86^ is as follows:

$$O_{ik}=\sum_{j=1}^{R}\;P_{ij}P_{kj}/\sqrt{\sum_{j=1}^{R}\;P_{ij}^{2}\sum_{j=1}^{R}\;P_{kj}^{2}}$$

where $O_{ik}$ represents the overlap index between species i and species k, $P_{ij}$ is the ratio of the number of individuals of species i in resource state j to the total number of individuals of species i, $P_{kj}$ is the ratio of the number of individuals of species k in resource state j to the total number of individuals of species k., and R is the total number of resource states. Resource states were divided into five intervals based on percentiles representing a gradient along a particular available resource. Available resources included TN, TP, DO, TSi, TC, etc.

**RF statistical analysis model:** RF is a machine learning algorithm based on ensemble learning proposed by Breiman and Cutler in 2001.^88^ This enhances the prediction capability and stability of the model by constructing many decision trees and using a voting mechanism to determine the final result. As one of the most widely applied and highly flexible machine learning algorithms, the core of RF lies in decision trees, fundamentally integrating multiple decision trees for learning.^89^ To ensure a more accurate measurement of variable importance, previous studies^90^ suggested that a higher number of trees helps reduce model randomness and overfitting risks, thereby improving model stability and predictive accuracy. In this study, we selected a large number of trees for each RF model (ntree: 10,000) and conducted 500 repeated evaluations of each tree error to assess the importance of various feature variables. Variable importance was calculated using increase in mean squared error (%IncMSE). Specifically, the larger this value, the more significant the contribution of the variable to the model's prediction. Additionally, we conducted a significance assessment of the variable importance based on a Permutation Test. This test generates a permutation distribution of importance scores by randomly shuffling variable values, and then compares the original variable importance with the scores in the permutation distribution to compute the p-value. A p-value below the significance level (e.g., 0.05) indicates that the variable significantly influences the model’s predictions. The permutation test does not require any specific distributional relationships between the variables and response variables, making it suitable for high-dimensional data analysis.^91^^,^^91^^,^^92^

**Unsupervised hierarchical clustering algorithm:** Hierarchical clustering is an unsupervised machine learning clustering method primarily used to group samples in a dataset based on similarity. It constructs a tree-like structure by progressively merging the most similar samples or clusters, thereby achieving hierarchical clustering of different types of samples. This method does not require a predefined number of clusters, allowing it to adapt flexibly to datasets of varying scales and distributions, thereby avoiding poor clustering results caused by improper cluster number selection. Additionally, it does not rely on the selection of initial centroids, thereby reducing the algorithm's sensitivity to initial conditions and preventing biases in the clustering results owing to inappropriate initial point selection.^93^^,^^94^ The silhouette coefficient is a method for quantifying the quality of the clustering results. Its value varies between -1 and 1. Higher value indicated better clustering performance. Silhouette coefficient can effectively evaluate clustering effectiveness and determine the optimal number of clusters.^95^ In this study, the optimal number of clusters was determined by the silhouette coefficient, and differential analysis was conducted on the clustered environmental factors based on the magnitude of each indicator after clustering. To further explore the characteristics of variation in species richness among different sites and the driving role of key environmental factors, a RF model was used to identify the key environmental factors with significant impacts on species richness. An unsupervised agglomerative hierarchical clustering method was applied to analyze the feature vectors of the 26 sites, and the Kruskal–Wallis test was used to validate the differences among different clusters.^44^ All analyses and plots were performed using R software (v.4.4.1) and Origin 2024.

### Quantification and statistical analysis

Ecological metrics, including species richness, niche breadth (Levins' formula), and niche overlap (Pianka's formula), were calculated to characterize community structure. A Random Forest regression model was utilized to evaluate variable importance via the increase in mean squared error (%IncMSE). Unsupervised hierarchical clustering, optimized via silhouette coefficients, was employed for ecological zoning, with all statistical analyses conducted in R (v.4.4.1).

### Additional resources

The supplementary datasets supporting this study, including the names of the 26 sampling locations across the main stream and tributaries , as well as the complete taxonomic list of the identified phytoplankton species, are available in the Appendix.
