# Supplementary material for: Identification of key environmental factors and intelligent ecological zoning in the upper Yangtze basin
Source: iScience. 2026 May 20;29(6):116001. doi: 10.1016/j.isci.2026.116001 (PMC13214539; doi:10.1016/j.isci.2026.116001)
Supplement: Document S1. Tables S1 and S2 [file mmc1.pdf]

iScience, Volume *29*

Supplemental information

Identification of key environmental factors and intelligent ecological zoning in the upper Yangtze basin

Wentong Wang, Peng Hu, Yunzhong Jiang, Baolong Zhao, Qin Yang, Qinghui Zeng, and Huasen Lu

Table S1. Summary of sampling sites Related to Figure 1

Sampling point	Name	Sampling point	Name
M1	Zhimenda	T4	Hengjiang
M2	Batang	M12	Yibin Upper
T1	Guxue	T4	Minjiang
M3	Benzilan	M13	Yibin Lower
M4	Shigu	M14	Sankuaishi
M5	Ahai	M15	Jiangan
M6	Zhongjiang	M16	Naxi
M7	Panzhihua	M17	Luzhou Upper
T3	Tongzilin	T5	Tuojiang
M8	Sanduizi	M18	Luzhou Lower
M9	Wudongde	M19	Hejiang
M10	Xiluodu	T6	Chishui
M11	Xiangjiaba	M20	Zhutuo

Table S2. Phytoplankton species list in the study area Related to Figure 2

Phylum	Genus	Species	Phylum	Genus	Species
	Microcystis	*Microcystis* sp.		***Chlamydomonas***	*Chlamydomonas* sp.
	Merismopedia	*Merismopedia* sp.		***Phacotus***	*Phacotus* sp.
	Rhabdogloea	*Rhabdogloea* sp.		***Chlorogonium***	*Chlorogonium* sp.
	Dactylococcopsis	*Dactylococcopsis* sp.		***Pandorina***	*Pandorina morum*
	Lyngbya	*Lyngbya* sp.		***Schroederia***	*Schroederia spiralis*
**Cyanophyta**	***Planktothrix***	*Planktothrix* sp.		***Chodatella***	*Chodatella* sp.
		Planktothrix spiroides		***Franceia***	*Franceia ovalis*
	Oscillatoria	*Oscillatoria* sp.		***Tetraëdron***	*Tetraëdron trilobulatum*
	Limnothrix	*Limnothrix* sp.			*Tetraëdron minimum*
	Pseudanabaena	*Pseudanabaena* sp.		***Ankistrodesmus***	*Ankistrodesmus* sp.
	Dolichospermum	*Dolichospermum* sp.		***Selenastrum***	*Selenastrum* sp.
	Cylindrospermopsis	*Cylindrospermopsis* sp.		***Kirchneriella***	*Kirchneriella* sp.
	Melosira	*Melosira* sp.		***Sphaercystis***	*Sphaercystis* sp.
		Melosira granulata		***Oocystis***	*Oocystis* sp.
		Melosira varians		***Nephrocytium***	*Nephrocytium* sp.
	Skeletonema	*Skeletonema* sp.	**Chlorophyta**	***Dictyosphaerium***	*Dictyosphaerium* sp.
	Coscinodiscus	*Coscinodiscus* sp.		***Scenedesmus***	*Scenedesmus* sp.
	Cyclotella	*Cyclotella* sp.			*Scenedesmus bijugus*
	Attheya	*Attheya* sp.			*Scenedesmus quadricauda*
	Diatoma	*Diatoma* sp.			*Scenedesmus dimorphus*
	Meridion	*Meridion* sp.		***Crucigenia***	*Crucigenia apiculata*
	Ceratoneis	*Ceratoneis* sp.			*Crucigenia tetrapedia*
	Fragilaria	*Fragilaria* sp.			*Crucigenia quadrata*
	Synedra	*Synedra* sp.		***Actinastrum***	*Actinastrum* sp.
		Synedra ulna		***Coelastrum***	*Coelastrum* sp.
	Asterionella	*Asterionella* sp.			*Coelastrum reticulatum*
	Eunotia	*Eunotia* sp.		***Closterium***	*Closterium gracile*
**Bacillariophyta**	***Frustulia***	*Frustulia* sp.		***Cosmarium***	*Cosmarium* sp.
	Gyrosigma	*Gyrosigma* sp.		***Planctonema***	*Planctonema* sp.
	Diploneis	*Diploneis* sp.		***Spirogyra***	*Spirogyra* sp.
	Navicula	*Navicula* sp.		***Mougeotia***	*Mougeotia* sp.
	Pinnularia	*Pinnularia* sp.		***Chroomonas***	*Chroomonas* sp.
	Amphiprora	*Amphiprora* sp.	**Cryptophyta**		*Chroomonas acuta*
	Amphora	*Amphora* sp.		***Cryptomonas***	*Cryptomonas* sp.
	Cymbella	*Cymbella* sp.			*Cryptomonas ovata*
	Gomphonema	*Gomphonema* sp.		***Euglena***	*Euglena* sp.
	Achnanthes	*Achnanthes* sp.	**Euglenophyta**	***Phacus***	*Phacus* sp.
	Rhoicosphenia	*Rhoicosphenia curvata*		***Trachelomonas***	*Trachelomonas* sp.
	Cocconeis	*Cocconeis* sp.		***Peridinium***	*Peridinium* sp.
	Nitzschia	*Nitzschia* sp.	**Dinophyta**	***Gymnodinium***	*Gymnodinium* sp.
		Nitzschia paradoxa		***Ceratium***	*Ceratium* sp.
	Surirella	*Surirella* sp.		***Dinobryon***	*Dinobryon* sp.
		Surirella capronii	**Chrysophyta**	***Kephyrion***	*Kephyrion* sp.
**Xanthophyta**	***Gonyostomum***	*Gonyostomum* sp.		***Chrysococcus***	*Chrysococcus* sp.
				Synura	*Synura* sp.
